# Insufficient access to oral paediatric medicines in Ghana: A descriptive study

**DOI:** 10.1186/s12913-016-1459-6

**Published:** 2016-06-17

**Authors:** Daniel N A Ankrah, Joseph T Turkson, Edith B Boateng, Frank T T Amegavie, Elizabeth Bruce

**Affiliations:** Division of Pharmacoepidemiology & Clinical Pharmacology, Utrecht Institute for Pharmaceutical Sciences (UIPS), Utrecht, The Netherlands; Pharmacy Department, Korle-Bu Teaching Hospital, Korle-Bu, Accra Ghana; Faculty of Pharmacy, University of Ghana, Legon, Accra Ghana

**Keywords:** Extemporaneous formulation, Paediatric medicines, Off-label use, Ghana

## Abstract

**Background:**

Among the most vulnerable people in society are children and this is especially so in their access to health care Off-label prescription of paediatric medicines is known to be associated with safety outcomes some of which may be serious. This study identifies frequently prescribed children’s medicines that are not readily available in Ghana and are prepared extemporaneously.

**Method:**

All prescriptions for extemporaneous oral preparations for children presented to the local production unit of the Korle-Bu Teaching Hospital from November, 2013 were eligible for the study. Information from such prescriptions was recorded in a systematic format. Presence of the prescribed medicine on the World Health Organization Children’s Medicine List was ascertained in addition to the anatomical and therapeutic classification code. The registration of the prescribed medicine for paediatric use by the Food and Drugs Authority, Ghana was also checked. Descriptive statistics of the data was presented.

**Results:**

In all 622 prescriptions for 35 different paediatric formulations were served. Prescriptions from several health facilities including government hospitals (6.6 %, *N* = 622), private hospitals (2.4 %, *N* = 622) and the University of Ghana hospital (1.1 %, *N* = 622) were all honoured. Some of the prescribed medicines (Baclofen, Clonazepam, Hydroxyurea and Lamotrigine) were neither on the World Health Organization Children’s Medicine list nor registered with the Food and Drugs Authority, Ghana. Most prescribed medicines (88.6 %, *N* = 35) were for non-communicable diseases.

**Conclusion:**

Paediatric prescriptions including off-label medicines are prescribed and formulated extemporaneously in this setting. Steps should be taken to improve access and monitor benefit-risk profiles of paediatric medicines in order to improve treatment outcomes among children.

## Background

Children are among the most vulnerable people in society, and concerted efforts are being made to increase their access to health care [1]. In times of illness, children need the appropriate medicines at the right time. Such medicines should be safe, efficacious, affordable and cost-effective.

Most adult oral medicines exist as tablets, capsules or caplets while most paediatric formulations are available as solutions, syrups, suspensions or granules for suspension. Adult medicines that may be used by children are not always readily available as paediatric formulations. For such medicines the best option is an extemporaneous formulation. It is on record that off-label use of medicines in children is associated with adverse reactions [2].

Extemporaneous preparations are available only in limited hospital pharmacies in Ghana, although most hospital pharmacies are superintended by pharmacists capable of producing such formulations. No private independent facilities produce individualised extemporaneous preparations in the country to date. The resultant pressure on the few existing facilities producing such formulations is extreme. In the end, it is parents or guardians of affected children who endure the most of such inadequacies by searching for these limited pharmacies because there are no arrangements for medicine pick-ups. This may lead to late treatment initiation among new patients and non-persistence among those already on treatment.

In Ghana, there are no guidelines for paediatric extemporaneous preparations, in contrast to places like Australia [3], for instance. There is no particular mechanism on how the price of such products should be calculated. To make matters worse, such products are not represented on the national health insurance medicines list, and patients must pay for these medicines out-of-pocket.

Ghana has an essential medicines list, which was adapted for use from the WHO Essential Medicines list [4]. The Ministry of Health Ghana under the Ghana National Drugs Programme (GNDP) has accepted the WHO priority medicines list for children and maternal health as a working document [5]. Having these medicine lists is necessary but not sufficient to guarantee treatment. Policy makers must ensure that systems are in place to provide the required medicines in the medicines list. Although extemporaneous formularies are difficult to obtain, Pharminfotech (New Zealand) has a formulary that is electronically accessible and free of charge [6].

This study aimed to identify frequently prescribed children’s medicines that are not readily available as manufactured preparations in Ghana and are thus prepared extemporaneously.

## Methods

### Setting

The study was carried out at the local production unit (LPU) of the Korle-Bu Teaching Hospital (KBTH) in Accra, Ghana. KBTH is a referral hospital with a 2000-bed capacity. The LPU is the arm of the Pharmacy Department of the hospital responsible for various extemporaneous formulations. Approximately 50 patients receive extemporaneous preparations from the LPU every week. These are predominantly in the form of oral suspensions and syrups. Preparations are formulated here with guidance from the Pharminfotech [6] online formulary.

Most of the prescriptions were from the hospital’s Child Health Sub-Budget Management Centre (sub-BMC) and the children’s unit of the National Cardio-Thoracic Centre (NCTC). The rest of the prescriptions were from other hospitals in the country.

### Participants and data collection

All prescriptions for extemporaneous oral preparations for children presented to the LPU from November 2013 were eligible for the study. Information from such prescriptions was recorded in a systematic format. The date, treatment centre, patient’s name and sex, age, name of preparation, and parent/caregiver-reported morbidity were captured prospectively. The data were anonymized for this study. Parent/caregiver reported morbidity was recorded because we did not use patient’s health records. Data capture continued until no new prescriptions were received for 14 continuous days. Patients who came for the same medication with different prescriptions on different occasions were captured more than once. However, those who came for refills on the same prescription form were only captured once.

### Ethics statement

This operational research was approved by the Korle-Bu Teaching Hospital’s Management as part of the Programme of Work of the Pharmacy Department to determine prescriptions patterns of paediatric medicines and to improve forecasting for planning purposes. The activity is conducted periodically to update the hospital formulary. The study is about prescribing practices in the hospital and poses less than minimum risk to patients. Because of these, there was no need for ethical approval after management’s approval.

### Analysis

The presence of the prescribed medicine on the WHO Children’s Medicines List was ascertained. Registration of the prescribed medicine for paediatric use by the Food and Drugs Authority (FDA) Ghana was also determined. The anatomical and therapeutic chemical (ATC) classification by the WHO [7] was also identified. Descriptive statistics of the data are presented using Stata intercooled version 12 (Stata Corp LP, College Station, TX, USA).

## Results

There were 622 individual prescriptions for extemporaneous preparations that were submitted during the study period. The highest proportion of prescriptions (0.35, 217/622) was in the first month of the study. This amount decreased gradually with time. Prescriptions from the Child Health sub-BMC and the NCTC amounted to 87.1 % of all requests for paediatric formulations, as would be expected given their association with the LPU. One prescription from Tamale Teaching Hospital, the most distant treatment site from the LPU (a distance of about 616 km from the study site) was dispensed. There were 7 (1.1 %) prescriptions from the University of Ghana hospital (Table 1). Prescriptions dispensed involved those for neonates, infants and children below 9 years.

**Table 1:** Characteristics at baseline

Characteristic	Frequency (*N* = 622)	Percentage frequency	Average distance from dispensary (km) ^b^
Period			
November 2013	217	35.0	Not applicable (N/A)
December 2013	182	29.2	N/A
January 2014	106	17.0	N/A
February 2014	117	18.8	N/A
Sex			
Male	272	43.7	N/A
Female	350	56.3	N/A
Hospitals			
Child Health Department	293	47.1	0
NCTC^a^	249	40.0	0
Government Hospitals	41	6.6	72
37 Military Hospital	16	2.6	12
Private Hospitals/Clinics	15	2.4	15
Tamale Teaching Hospital	1	0.2	616
University of Ghana Hospital	7	1.1	18
Age groups			
≤ 1 month	37	6.0	N/A
> 1 month–12 months	167	26.8	N/A
> 12 months – 60 months	250	40.2	N/A
> 60 months	25	4.0	N/A
Missing	143	23.0	N/A

^a^National Cardio-Thoracic Centre. ^b^Average distance was used because of grouped data

The most frequently reported morbidities were cardiovascular diseases, anaemia and seizures. Table 2 shows the list of drugs and their ATC classifications. Furosemide (31.2 %, *N* = 622), spironolactone (28.1 %, *N* = 622), folic acid (9.0 %, *N* = 622), propranolol (8.0 %, *N* = 622) and clonazepam (3.2 %, *N* = 622) suspensions were the top five most dispensed medicines. Some medicines were also dispensed, which were neither registered by the FDA Ghana nor present on the WHO Children Medicines List 2010 [1]. These included baclofen (1.9 %, *N* = 622), clonazepam (3.2 %, *N* = 622), hydroxyurea (2.6 %, *N* = 622) and lamotrigine (1.0 %, *N* = 622).

**Table 2:** Prescribed medicines at baseline with ATC codes

Suspension/syrup	Number of prescriptions (%) (*N* = 622)	Prescription ever registered in Ghana	Prescription registered for oral paediatric use	Availability on children EML^a^	ATC code [7]
Baclofen	12 (1.9)	No	-^b^	No	M03BX01
Captopril	15 (2.4)	Yes	No	No	C09AA01
Clonazepam	20 (3.2)	No	-	No	N03AE01
Digoxin	2 (0.3)	Yes	No	Yes	C01AA05
Domperidone	2 (0.3)	Yes	Yes	No	A03FA03
Enalapril	2 (0.3)	Yes	No	Yes	C09AA02
Folic acid	56 (9.0)	Yes	Yes	Yes	B03BB01
Furosemide	194 (31.2)	Yes	No	Yes	C03CA01
Hydroxyurea	16 (2.6)	No	-	No	L01XX05
Levetiracetam	2 (0.3)	Yes	No	No	N03AX14
Lamotrigine	6 (1.0)	No	-	No	N03AX09
Lisinopril	3 (0.5)	Yes	No	No	C09AA04
Metoclopramide	2 (0.3)	Yes	Yes	Yes	A03FA01
Nalidixic Acid	2 (0.3)	Yes	No	No	J01MB02
Nifedipine	4 (0.6)	Yes	No	No	C08GA02
Nitrofurantoin	3 (0.5)	Yes		Yes	J01XE01
Phenobarbitone	14 (2.3)	Yes	Yes	Yes	N03AA02
Prednisolone	2 (0.3)	Yes	Yes	Yes	H02AB06
Propranolol	50 (8.0)	Yes	Yes	Yes	C07BA05
Pyrimethamine	3 (0.5)	No	-	Yes	P01BD01
Ranitidine	2 (0.3)	Yes		Yes	A02BA02
Risperidone	7 (1.1)	Yes	No	No	N05AX10
Sildenafil citrate	13 (2.1)	Yes	No	No	G04BE03
Spironolactone	175 (28.1)	Yes	No	Yes	C03DA01
Sulfadiazine	3 (0.5)	No	-	Yes	J01EC02
Topiramate	3 (0.5)	Yes	-	No	N03AX12
Others	9 (1.4)	-	-	-	-

^a^WHO Model Formulary for Children 2010. ^b^There was no information on product

## Discussion

This study shows that a number of prescribed medicines for paediatric use are not readily available on the Ghanaian market, and are formulated extemporaneously using adult tablets. The formulations are prepared only at restricted facilities and care givers have to travel long distances for their medicines. Furthermore, the study reveals that some of the prescribed medicines are neither present on the WHO priority medicines list for children [1] nor registered with the Food and Drugs Authority in the country. The study also reveals that most of the formulated medicines are for management of non-communicable disease.

This article emphasizes the need to provide alternative and appropriate formulations for infants and children where manufactured formulations are not readily available. Our findings also suggest that pharmacists should learn the techniques involved in preparation of extemporaneous products to make life easier for patients and their caregivers without compromising safety.

There is an urgent need for immediate assessment and harmonization of the paediatric extemporaneous preparation situation in Ghana. Pharmacists should be trained in all regional hospitals and encouraged to train pharmacists in other practice settings throughout their regions. There should be proper evaluation of prescription patterns and provision of basic accoutrements for effective production and dispensing. Pharmacists involved with such formulations should be well motivated to deliver medication promptly and avoid excessive patient waiting times.

About 90 % of formulated medicines are indicated in management of non-communicable diseases. According to the WHO, congenital anomalies and other non-communicable diseases contribute to 7 % of all deaths among infants and children aged below 5 years [8]. This underscores the importance of these formulations or a search for appropriate pre-formulated alternatives. Policy makers need to make an immediate intervention to bring this situation under control.

Reports from UNICEF indicate that in 2012 about 44 % of all deaths in children younger than 5 years occurred in neonates, but most of these deaths were preventable [9]. Although malnutrition has been cited as one of the underlying reasons for these deaths, unavailability of the appropriate prescribed medicine could also be a contributing factor [9].

The fact that teaching hospitals, university hospitals, government hospitals, private hospitals, and clinics are all prescribing these paediatric formulations indicates the extent to which they have become essential. The high number of prescriptions at the KBTH Child Health sub-BMC and the NCTC (both referral centres) in our study clearly shows that these medicines are mostly prescribed by specialists and/or consultants. This calls for re-evaluation of the national essential medicines list.

The use of unlicensed or off-label medicines among paediatric patients has been reported to be significantly associated with an increased risk of developing an adverse drug reaction [2]. Furthermore, among some hospitalised children, up to 90 % of medicines are prescribed off-label [10–14]. For those medicines that are neither present on the Children’s Medicines list nor registered with the FDA Ghana for paediatric use, measures should be taken to rectify the situation and encourage proper monitoring of benefit-risk profiles of such medicines among this subgroup.

The use of specially formulated medicines is not limited to low and medium income countries like Ghana. In the United Kingdom (UK) these products are popularly called “specials” or “special order product” [15]. Most specials are unlicensed formulations of a licensed medicine [15]. The difference is that such facilities are well established, accessible, and regulated. Special formulations are available in New Zealand [6] and Australia [3] where well established formularies are used.

Our study captured only those prescriptions that were filled. If prescriptions that were not dispensed had also been captured, this would have been more interesting. This would further emphasize the magnitude of the unmet need for paediatric formulations in Ghana. There were missing values for patient’s age. This occurred because data were captured from prescription forms, some of which did not indicate the patient’s age. This information would have allowed the most at-risk age group for these formulations to be identified.

## Conclusion

In conclusion, the non-availability of pre-formulated paediatric medicines in Ghana has resulted in the extemporaneous formulation of such medicines using adult tablets. Only limited facilities provide this service. Ghana, and other countries facing this challenge could learn from the UK by encouraging the establishment of facilities for the preparation of specials at strategic positions throughout the country and regulate them. The current situation poses a challenge to care givers who have to travel long distance to procure these medicines. Some of these medicines are neither present on the WHO Children’s Medicines list nor registered with the FDA Ghana, emphasizing the possibility of off-label use of medicines. Steps should be taken by policy makers to involve more facilities in the preparation of these medicines while the search for pre-formulated forms continue.

### Abbreviations

ATC, anatomical and therapeutic classification; BMC, budget management committee; FDA, food and drug authority, Ghana; GNDP, Ghana national drug program; KBTH, Korle-Bu teaching hospital; LPU, local production unit; MOH, ministry of health; NCTC, national cardio-thoracic centre; UNICEF, United Nations children’s fund; WHO, world health organization.

## References

[1] WHO Model Formulary for Children (2010). Based on the Second Model List for Essential Medicines for Children 2009.

[2] Neubert A, Dormann H, Weiss J, Egger T, Criegee-Rieck M, Rascher W (2004). The impact of unlicensed and off-label drug use on adverse drug reactions in paediatric patients. Drug Saf.

[3] Department of Health, Pharmaceutical Benefits Scheme, Australia. Available: http://www.pbs.gov.au/info/healthpro/explanatory-notes/section1/Section_1_9_Explanatory_Notes. Accessed 25 Oct 2014.

[4] Sinclair D (2013). Integrating Global and National Knowledge to Select Medicines for Children: The Ghana National Drugs Programme. PLOS Medicine..

[5] Priority medicines for mothers and children 2011. World Health Organization. Department of essential medicines and pharmaceutical policies. Available: http://www.who.int/medicines/publications/A4prioritymedicines.pdf. Accessed 2 Aug 2014

[6] Pharminfotech. Available:http://www.pharminfotech.co.nz. Accessed 20 Aug 2013.

[7] ATC/DDD Index 2014. WHO Collaborating Centre for Drug Statistics Methodology. Norwegian Institute of Public Health. Available: http://www.whocc.no/atc_ddd_index/. Accessed 26 Nov 2014.

[8] Committing to child survival: A promise renewed. Progress report 2013. Available: http://www.unicef.org/lac/Committing_to_Child_Survival_APR_9_Sept_2013.pdf. Accessed 1 Dec 2014.

[9] Causes of death among children under five years 2013. Available: http://www.who.int/gho/child_health/mortality/causes/en/. Accessed 1 Dec 2014.

[10] McIntyre J, Conroy S, Avery A (2000). Unlicensed and off label prescribing of drugs in general practice. Arch Dis Child.

[11] Pandolfini C, Impicciatore P, Provasi D (2002). Off-label use of drugs in Italy: a prospective, observational and multicentre study. Acta Paediatr.

[12] Conroy S, Choonara I, Impicciatore P (2000). Survey of unlicensed and offlabel drug use in paediatric wards in European countries. BMJ.

[13] Cuzzolin L, Zaccaron A, Fanos V (2003). Unlicensed and off-label uses of drugs in paediatrics: a review of the literature. Fundam Clin Pharmacol.

[14] Stephenson T (2001). Medicines for children—the last century and the next. Arch Dis Child.

[15] Chaplin S (2014). How drug tariff specials have reduced prescribing costs. Prescriber.

